# Refined Multiscale Entropy Analysis of Wrist Pulse for Gender Difference in Traditional Chinese Medicine among Young Individuals

**DOI:** 10.1155/2022/7285312

**Published:** 2022-02-08

**Authors:** Huaxing Xu, Qia Wang, Xiaobo Mao, Zhigang Shang, Yuping Zhao, Luqi Huang

**Affiliations:** ^1^School of Electrical Engineering, Zhengzhou University, Zhengzhou 450001, Henan, China; ^2^Institute of Quantitative and Technological Economics, Chinese Academy of Social Sciences, Beijing 100732, China; ^3^China Academy of Chinese Medical Sciences, Beijing 100020, China

## Abstract

Pulse signal analysis plays an important role in promoting the objectification of traditional Chinese medicine (TCM). Like electrocardiogram (ECG) signals, wrist pulse signals are mainly caused by cardiac activities and are valuable in analyzing cardiac diseases. A large number of studies have reported ECG signals can distinguish gender characteristics of normal healthy subjects using entropy complexity measures, consistently showing more complexity in females than males. No research up to date, however, has been found on examining gender differences with wrist pulse signals of healthy subjects on entropy complexity measures. This paper is aimed to fill in the research gap, which could, in turn, provide a deeper insight into the pulse signal and might identify potential differences between ECG signals and pulse signals. In particular, several complementary entropy measures with corresponding refined composite multiscale versions are established to perform the analysis for the filtered TCM pulse signals. Experimental results reveal that regardless of entropy measures used, there is no statistically significant gender difference in terms of entropy complexity, indicating that the pulse signal holds less gender characteristics than the ECG signal. In view of these findings, wrist pulse signals could be likely to provide different pieces of information to ECG signals. The present study is the first to quantitatively evaluate gender differences in healthy pulse signals with measures of entropy complexity and more importantly; we expect this study could make contribution to the ongoing pulse intelligent diagnosis and objective analysis, further facilitating the modernization of TCM pulse diagnosis.

## 1. Introduction

In general, there are four major diagnostic methods of traditional Chinese medicine (TCM), i.e., looking, listening, asking, and feeling the pulse. Among them, pulse diagnosis refers to placing the doctor's three fingers on the wrist radial artery to analyze the health condition [1–4]. For thousands of years, pulse diagnosis has played an indispensable role in TCM as well as in traditional Indian/Korean medicine. Even in today's disease diagnosis, pulse diagnosis is still very competitive due to its convenient, inexpensive, and noninvasive advantages. Furthermore, TCM has also been increasingly adopted in the West by medical practitioners as a supplementary and alternative medical treatment [5].

The basic principle behind wrist pulse-based diagnosis relies on the fact that when blood flows through the organs of the whole body, the disease of which will be eventually reflected in the pulse fluctuation pattern [6]. It is held in TCM theory that pulse conditions are closely tied to the heart beating, blood patency and adequacy, and deficiency of Qi and blood [2, 7]. However, the practice of TCM pulse diagnosis is highly subjective, extremely depending on the experience of the practitioners which usually require years of training. In this case, diagnosis results may be not so objective and reliable. To overcome these limitations, objective analysis and interpretation of the wrist pulse signal, known as computational pulse signal analysis, has been developed in the last few decades [8–13].

Being a physiological signal, the wrist pulse signal is the same as the electrocardiogram (ECG) signal, mainly driven by cardiac contraction and relaxation [14, 15]. Analysis approaches derived from the nonlinear dynamic system have been extensively explored in the study of the characteristics of cardiovascular dynamics [16]. For healthy individuals, a large number of studies have reported that there is a significant gender difference in ECG signals in terms of various complexity measures, such as [17–21]. Since pulse signals can also reflect the heart condition and the vascular system, it is a natural and reasonable assumption for the pulse signal obtained from the subject's superficial artery also to be an indicator of gender differences. On the other hand, gender differences are also considered as an important intrinsic factor in wrist pulse assessment in TCM.

For physiological signal processing, analysis methods can be linear or nonlinear. Several previous works have already applied linear methods in time-domain [22, 23] and frequency-domain [23] to investigate the gender difference in the pulse signal from healthy people. Based on their findings, it has been confirmed that gender differences can be characterized by some features in the pulse signal. Nevertheless, linear analysis techniques may provide less information about the integrated dynamic characteristics of pulse signals. For this reason, it is imperative to examine the gender dependency of the pulse signal with widely used nonlinear dynamics indices.

As one of the nonlinear dynamics indices, some nonlinear complexity entropies, such as approximate entropy [24] or sample entropy [11, 25, 26], have been proposed for computational pulse diagnosis. As expected, signal entropy is distinct between normal people and patients with respect to complexity measures. In view of this, the available studies are primarily focusing on extracting the entropy feature to perform classification on different patients or healthy people and patients. To our best knowledge, there is no report on distinguishing the gender of healthy subjects based on entropy complexity measures. This paper makes an attempt to fill this gap. Specifically, we utilize four kinds of complementary entropy statistics to seek gender difference characteristics embedded in the pulse signal of normal people, which will probably provide additional valuable information related to dynamical changes of the pulse signal. Furthermore, the study presented in the paper might identify potential differences between ECG signals and pulse signals in terms of entropy complexity.

Entropy, as a complexity measure, has been widely applied for different time series analyses, such as industrial fault diagnostic systems [27] and biological signal analyses [28]. Over the years, many different entropy algorithms have been introduced in the literature. For a more comprehensive analysis, our research covers two large families: one is based on Shannon's entropy and the other is based on embedding [29]. As far as entropy measures are concerned in the paper, sample entropy (SaEn) and its improved variant-fuzzy entropy (FuEn) belong to the former group, while permutation entropy (PeEn) [30] and dispersion entropy (DiEn) [31] are the representatives of the latter family [29]. For detailed comparative analysis, both single and multiscale entropy algorithms are implemented.

Approximate entropy (ApEn) is the first widely used entropy measure developed for nonlinear time series analysis [32]. It is however biased resulting from self-matching included when calculating the occurrences of similarity. To address this issue, SaEn is proposed as an extension of ApEn [33] and has been verified to be superior to it [34, 35]. Therefore, in this paper, we first choose the SaEn measure instead of the ApEn measure to analyze the pulse signal. Unfortunately, the SaEn performance is very sensitive to the tolerance since the hard threshold is used. FuEn with the smooth threshold function is then introduced to improve the robustness of SaEn [36, 37]. Irrespective of healthy people or patients, FuEn has not been applied to computational pulse signal analysis yet. This paper also tries to evaluate the performance of the FuEn measure for healthy people.

Despite the popularity of SaEn or FuEn, there are still some problems. First, both ApEn and FuEn ignore the temporal order of the values in a time series. In addition, SaEn, including FuEn, is sensitive to signal amplitude changes. Instead of calculating the entropy with respect to the amplitude, PeEn takes into count the analysis of ordinal patterns by estimating the relative frequencies in time series [30]. Since only ordinal patterns are considered, the amplitude of the signal is practically irrelevant, resulting in structural robustness to the noise. Furthermore, it has the quality of simplicity, robustness, and very high calculation efficiency. Therefore, permutation entropy is also used as an analysis index. On the other hand, PE solely considers the order of the amplitude values, and hence, some crucial information may be missed. To tackle these problems, recently Rostaghi and Azami [31] proposed a new method, termed dispersion entropy (DiEn), which also considers the mean value of amplitudes and differences between amplitude values. They show that the DiEn method considerably outperforms PeEn to discriminate different groups of each dataset. In addition, the computation time of DiEn is significantly less than that of PeEn. For a comprehensive comparison, we also include the DiEn measure to characterize ordinal patterns of the pulse signal.

In practice, time series derived from physiological and complex nonlinear dynamic systems contain multiple temporal scale structures [38]. It is found that traditional entropy measures, like SaEn, may lead to misleading results due to their single-scale-based measures. To prevent this, Costa et al. [38] propose a new entropy complexity measure, known as multiscale entropy measure (MSE), which takes different scales of a time series into account. To be specific, the multiple scales of input data are first derived and the associated entropy measures are subsequently calculated for each scale separately. Based on this novel idea, a large number of corresponding MSEs have been successfully applied in the biomedical research field [39–42]. It is worth noting that, compared to other physiological signals, pulse data recording is shorter (several thousand in general). As a result, for traditional MSE algorithms, the coarse-grained procedures can reduce the length of a time series, which may induce an inaccurate estimation of entropy or undefined entropy. Wu et al. [43] have demonstrated that the refined composite MSE (RCMSE) algorithm, which is independent of the data length, is more reliable and better than traditional MSE algorithms. This paper, therefore, incorporates the refined composite multiscale architecture [44–46] into the used four entropy measures.

The primary objective of this work is to comprehensively and systematically examine gender differences of wrist pulse signals from healthy people in terms of various entropy measures with a refined composite multiscale framework. To do this, the remainder of the paper is organized as follows.  Section 2 describes the acquisition of wrist pulse data and the necessary preprocessing process for pulse signals, followed by the brief review of four kinds of entropy measures and the refined composite multiscale framework in Section 3. Experimental results and analysis with different entropies and scales are presented in Section 4.  Section 5 compares the difference between the pulse and ECG signals in distinguishing the healthy people's gender, investigates other existing related pulse work, and at last states limitations of our work. The conclusion is finally drawn in Section 6. We believe that in terms of entropy measures, new findings regarding gender differences in our work may advance guidelines for improved pulse diagnosis or analysis and further provide some insights into the modernization of traditional pulse diagnosis.

## 2. Materials and Preliminaries

### 2.1. Research Subjects

The volunteers are all adult college students of Zhengzhou University. All participants have good health without clinical evidence of any disease. Anyone taking any medicine or unable to complete pulse measurement is excluded, especially women who are in menstruation. In order to measure the wrist pulse signal as accurately as possible and prevent interference from other factors, participants are informed not to consume caffeine or alcohol and vigorous exercise the day before the pulse-taking. The same activities as well as eating and smoking are not allowed within 2 hours before the test.

In order to ensure data quality and balance, from the collected data, a total of 200 right-handers are equally divided into two groups of 100 males and 100 females to study gender differences.  Table 1 lists their physical conditions including height, age, and weight expressed as mean ± standard deviation (SD). From the table, we can figure out that the female group is lower in weight and height than the male group, but almost the same in age.

### 2.2. Wrist Pulse Signal Acquisition

**Figure 1:**
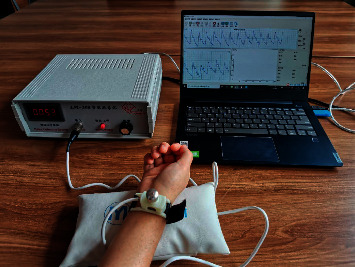
Wrist pulse signal acquisition.

By far, a number of sensors, such as pressure, photoelectric, and ultrasonic sensors, have been developed for acquiring pressure pulse signals. Compared with photoelectricity and Doppler ultrasonic sensors, the operating principle of the pressure sensor is more consistent with the TCM theory [47]. In this study, the ZM-300 intelligent TCM pulse pressure detector (made by Shanghai University of Traditional Chinese Medicine, Shanghai, China) is used to collect the wrist ulna pulse signal (at the chi position) in both the left arm and right arm, respectively. The pulse signal acquisition system used in this work is illustrated in Figure 1.

### 2.3. Experimental Setup

This study is conducted in a very quiet room and follows relevant guidelines and regulations. All subjects rested for 10 minutes to stabilize the resting heart rate prior to taking the pulse. During the signal acquisition process, subjects sit in a relaxed and comfortable way and keep their back straight. Pulse collectors have been trained by professional doctors and wrap the pressure sensor in the radial arteries of the wrist in order of right arm to the left arm.

For each volunteer, pulses are monitored for 10 seconds at a sampling rate of 200 Hz; therefore, 2000 samples in total are captured. Pulse manifestation signals under different pressures are obtained by imposing 6 different pressures on each hand and the optimal pulse signal is picked for processing and analysis.

### 2.4. Pulse Signal Preprocessing

The sampling frequency we used is high enough to prevent pulse waveform distortion. However, the pulse signal is inevitably corrupted by background noise and baseline drift. Before subsequent signal processing, these interferences need to be eliminated to obtain a clean pulse signal. For the preprocessing, we adopt the robust signal preprocessing framework proposed in [48], which first denoises the pulse and then removes baseline drift with a wavelet-based cascaded adaptive filter [49].

Generally, the frequency range of the pulse signal is between 0 Hz and 10 Hz, not exceeding 40 Hz [50]. The background noise is mainly caused by high-frequency interference such as environment disturbance and electricity interruption. Therefore, the noise can be filtered out by a low-pass filter with a cut-off frequency of 40 Hz. As for the baseline drift, various solutions have been proposed to correct it in physiological signals, among which the wavelet-based cascaded adaptive filter method stands out [49]. In the filter, the pulse signal is decomposed and its baseline drift level is detected by computing its energy ratio. According to the given threshold, the pulse is then filtered with a discrete Meyer wavelet filter followed by cubic spline estimation.

**Figure 2:**
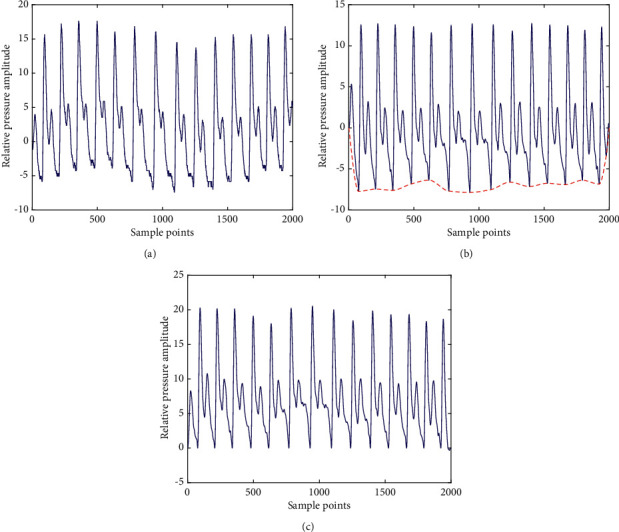
Pulse waveform preprocessing. (a) Original pulse signal. (b) Denoised pulse signal after a low-pass filter; the baseline of the signal is shown in black. (c) Baseline-removed pulse signal.

Figure 2 shows a snapshot of pulse waveform preprocessing. As we can see, the raw pulse waveform is greatly enhanced so that the accurate extraction of pulse interval can be assured in the following step.

After preprocessing, some pulse signals are excluded by visual inspection due to technical artifacts. Furthermore, we remove incomplete cycles and normalize the pulse further for ensuring precise and validated entropy computing [24].

## 3. Methods

Entropy measure is widely used to evaluate the complexity of physiological signals in many research fields. In this section, the basic ideas underlying the definition of these selected entropy measures are briefly reviewed; the reader is recommended to refer to [30, 37, 44, 45] and references therein for more details.

### 3.1. Sample Entropy Measure

For the wrist pulse signal *x*(*i*)(1 ≤ *i* ≤ *N*), given the embedding dimension *m* and tolerance *r*, the SaEn of time series can be computed as follows:

(1) First, construct the template vectors *X*_*i*_^*m*^(1 ≤ *i* ≤ *N* − *m*+1) as follows:
  (1) $$\begin{array}{c} X_{i}^{m}=\left\{x\left(i\right),x\left(i+1\right),\ldots ,x\left(i+m-1\right)\right\}. \end{array}$$
Vector sequences *X*_*i*_^*m*^ represent *m* consecutive *x*(*i*).
(2) Second, for each *X*_*i*_^*m*^, the distance between *X*_*i*_^*m*^ and *X*_*j*_^*m*^ is calculated by using the infinite norm, defined as
  (2) $$\begin{array}{c} d_{ij}^{m}=\underset{k=0,\ldots ,m-1}{\max}\left(\left|x\left(i+k\right)-x\left(j+k\right)\right|\right). \end{array}$$
(3) Third, with the Heaviside function Θ(*x*), the number of vector matches is counted as a tolerance *r* in the following way:
  (3) $$\begin{array}{c} C_{i}^{m}\left(r\right)=\frac{1}{N-m-1}\sum_{j=1,j\neq i}^{N-m}\Theta \left(r-d_{ij}^{m}\right), \end{array}$$
where *j* ≠ *i* excludes self-matches. Based on this, the probability that two vectors of length *m* match with tolerance *r* is then
  (4) $$\begin{array}{c} \phi^{m}\left(r\right)=\frac{1}{N-m}\sum_{i=1}^{N-m}\ln C_{i}^{m}\left(r\right). \end{array}$$
Similarly, repeat the above process for vectors of length *m*+1:
  (5) $$\begin{array}{c} C_{i}^{m+1}\left(r\right)=\frac{1}{N-m-1}\sum_{j=1,j\neq i}^{N-m}\Theta \left(r-d_{ij}^{m+1}\right),\\ \phi^{m+1}\left(r\right)=\frac{1}{N-m}\sum_{i=1}^{N-m}C_{i}^{m+1}\left(r\right). \end{array}$$
(4) Finally, the SaEn is calculated with the following equation:

(6) $$\begin{array}{c} \text{SaEn}\left(m,r,N\right)=\ln\left(\phi^{m}\left(r\right)\right)-\ln\left(\phi^{m+1}\left(r\right)\right). \end{array}$$

The calculation of SaEn requires two parameters to be determined in advance: (i) the embedding dimension *m*, describing the length of vectors to compare; and (ii) the tolerance threshold *r*, the distance threshold for two template vectors. The data length is preferred in the range between 10^*m*^ and 20^*m*^ [51]. The tolerance is usually recommended between 10% and 20% of the standard deviation *σ* of the signal's amplitudes [38]. In what follows the values of *m*=3 and *r*=0.15 *σ* have been used.

### 3.2. Fuzzy Entropy Measure

In SaEn, the pattern similarity is determined by the Heaviside function Θ(*d*_*ij*_^*m*^ − *r*) given in equation (3). To counter this discontinuity, FuEn employs a fuzzy membership function to compute the similarity degree between two patterns. Essentially, the computation of FuEn is only modified with a new distance measure.

Typically, the similarity degree is determined using a family of an exponentially decaying function:

(7) $$\begin{array}{c} D_{ij}^{m}\left(n,r\right)=\exp\left(-{\left(\frac{d_{ij}^{m}}{r}\right)}^{n}\right), \end{array}$$

where *n* defines the membership function shape. In this paper, we use *n*=2 as proposed in [36, 37]. Then, the equations for the match counts are

(8) $$\begin{array}{c} C_{i}^{m}\left(r\right)=\frac{1}{N-m-1}\sum_{j=1,j\neq i}^{N-m}D_{ij}^{m}\left(n,r\right),\\ C_{i}^{m+1}\left(r\right)=\frac{1}{N-m-1}\sum_{j=1,j\neq i}^{N-m}D_{ij}^{m+1}\left(n,r\right). \end{array}$$

Under given tolerance *r*, the matching probabilities of vectors of lengths *m* and *m*+1 are calculated the same as SaEn, and finally, FuEn is estimated as

(9) $$\begin{array}{c} \text{FuEn}\left(m,2,r,N\right)=\text{ln}\left(\phi^{m}\left(r\right)\right)-\text{ln}\left(\phi^{m+1}\left(r\right)\right). \end{array}$$

### 3.3. Permutation Entropy Measure

The concept of PeEn is to map a continuous time series onto a symbolic sequence, where the statistics of the symbolic sequences are called permutation entropy. The specific calculation process is as follows:

(1) First, for given embedding dimension *m*, the phase space *X*_*i*_^*m*^(1 ≤ *i* ≤ *N* − *m*+1) of a time series wrist pulse signal *x*(*i*)(1 ≤ *i* ≤ *N*) can be constructed as
  (10) $$\begin{array}{c} X_{i}^{m}=\left\{x\left(i\right),x\left(i+1\right),\ldots ,x\left(i+m-1\right)\right\}. \end{array}$$
Furthermore, each sequence *X*_*j*_^*m*^ is sorted in ascending order with a permutation pattern *π*_*i*_^*m*^ and there will be *m*! possible permutations *π* for an *m*− tuple vector.
(2) Based on the probabilities of all permutations, the PeEn is defined as follows:
  (11) $$\begin{array}{c} H_{p}\left(m\right)=-\sum_{i=1}^{m!}p\left(\pi_{i}^{m}\right)\ln\;p\left(\pi_{i}^{m}\right), \end{array}$$
where *p*(*π*_*i*_^*m*^) is calculated as
  (12) $$\begin{array}{c} p\left(\pi_{i}^{m}\right)=\frac{\#\left\{j|j=1,\ldots ,N-m+1;X_{j}^{m}\text{ has type }\pi_{i}^{m}\right\}}{N-m+1}. \end{array}$$
(3) It is clear that PeEn values are between in the range [0, log  *m*!]. For convenience, the normalized permutation entropy is computed as

(13) $$\begin{array}{c} 0\leq \text{PeEn}=\frac{H_{p}\left(m\right)}{\log\left(m!\right)}\leq 1. \end{array}$$

The maximum (minimum) value of PeEn is 1 (0), indicating that each ordinal pattern has the same probability or the time series is very regular. In brief, the smaller the value of PeEn, the more regular the time series.

The evaluation of the appropriate permutation distribution relies on the embedding dimension *m*. To achieve reliable statistics, PeEn requires that the length *N* of the time series satisfies *N* ≫ *m*! [52]. In practice, it is suggested to work with 3 ≤ *m* ≤ 7 [30]. In our study, we set *m*=3 in order to maintain consistency with the aforementioned two entropy measures.

### 3.4. Dispersion Entropy Measure

Dispersion entropy originates from permutation entropy and is also related to the embedding dimension *m* and the time delay. In practice, it is recommended *d*=1 [31]; thus, for clarity, the specific calculation process of DiEn is as follows:

(1) First, for the given pulse signal, employ the normal cumulative distribution function (NCDF) to map *x*(*i*) into *y*={*y*_1_, *y*_2_,…, *y*_*N*_} from 0 to 1. The new time series *y*_*j*_ is assigned each to an integer from 1 to *c* with the following linear algorithm:
  (14) $$\begin{array}{c} z_{j}^{c}=\text{round}\left(c^{*}y_{j}+0.5\right), \end{array}$$
where *z*_*j*_^*c*^ shows the *j*th member of the classified time series and *roun*  *d* is the integer function.
(2) Next, for given embedding dimension *m*, compute the phase space reconstruction *z*_*i*_^*m*,*c*^(1 ≤ *i* ≤ *N* − *m*+1) for the time series *z*_*j*_^*c*^,
  (15) $$\begin{array}{c} z_{i}^{m,c}=\left\{z_{i}^{c},z_{i+1}^{c},\ldots ,z_{i+\left(m-1\right)}^{c}\right\}. \end{array}$$
Each time series *z*_*i*_^*m*,*c*^ is mapped to a dispersion pattern *π*_*v*_0_,*v*_1_,…,*v*_*m*−1__, where *z*_*i*_^*c*^=*v*_0_, *z*_*i*+1_^*c*^=*v*_1_,…, *z*_*i*+(*m* − 1)_^*c*^=*v*_*m*−1_ and the number of all possible dispersion patterns is *c*^*m*^.
(3) For each of *c*^*m*^ potential dispersion patterns, the relative frequency is obtained as follows:
  (16) $$\begin{array}{c} p\left(\pi_{v_{0}\ldots v_{m-1}}\right)=\frac{\#\left\{i|i\leq N-\left(m-1\right)d,z_{i}^{m,c}\text{ has type }\pi_{v_{0}\ldots v_{m-1}}\right\}}{N-\left(m-1\right)d}. \end{array}$$
(4) Finally, based on Shannon's definition of entropy, the dispersion entropy is computed as

(17) $$\begin{array}{c} H_{d}\left(m\right)=-\sum_{\pi =1}^{c^{m}}p\left(\pi_{v_{0}v_{1}\ldots v_{m-1}}\right)\ln\left(p\left(\pi_{v_{0}v_{1}\ldots v_{m-1}}\right)\right). \end{array}$$

Clearly, DiEn values range in [0, log(*c*^*m*^)]. For convenience, the normalized dispersion entropy is computed as

(18) $$\begin{array}{c} 0\leq \text{DiEn}=\frac{H_{d}\left(m\right)}{\log\left(c^{m}\right)}\leq 1. \end{array}$$

For the practical purpose of DiEn, we choose *c*=6, just as recommended in [31]. Again, the embedding dimension *m* is set 3 as in the PeEn.

### 3.5. RCMSE Framework

Traditional MSE may produce incorrect entropy estimates and lead to uncertain entropy because coarse-grained processes will greatly reduce data length on a large scale. To address these drawbacks, the RCMSE algorithm is employed in this paper, with specific procedures summarized as follows:

(1) First, construct multiple coarse-graining series. For time scale *τ*, there are *k* coarse-grained time series *y*_*k*_^(*τ*)^={*y*_*k*,1_^(*τ*)^, *y*_*k*,2_^(*τ*)^,…, *y*_*k*,*p*_^(*τ*)^}, which is defined as follows:
  (19) $$\begin{array}{c} y_{k}^{\left(\tau \right)}=\frac{1}{\tau }\sum_{i=\left(j-1\right)\tau +k}^{j\tau +k-1}x_{i},\;1\leq j\leq \frac{N}{\tau },1\leq k\leq \tau . \end{array}$$
(2) For SaEn or FuEn, the number of matched vector Paris *n*_*k*,*τ*_^*m*+1^ and *n*_*k*,*τ*_^*m*^ is computed for all *τ* coarse-grained series. RCMSE is then defined as the logarithm of ration mean number of matched vector Paris [43, 44].
  (20) $$\begin{array}{c} \text{RCMSE}\left(x,\tau ,m,r\right)=-\ln\left(\frac{{\bar{n}}_{k,\tau }^{m+1}}{{\bar{n}}_{k,\tau }^{m}}\right)=-\ln\left(\frac{\sum_{k=1}^{\tau }n_{k,\tau }^{m+1}}{\sum_{k=1}^{\tau }n_{k,\tau }^{m}}\right). \end{array}$$
For calculating the matching template, using different distance metrics will produce corresponding refined composite multiscale entropy. In this paper, we refer to the refined composite multiscale version of SaEn and FuEn as RC_MSE_ and RC_MFE_ in respective order.
(3) For refined composite multiscale permutation entropy (abbreviated as RC_MPE_) [45] and refined composite multiscale permutation entropy (abbreviated as RC_MDE_) [46], the calculation is slightly different, which is computed as

(21) $$\begin{array}{c} {\text{RC}}_{MP\left(D\right)E}\left(x,\tau ,m,L\right)=-\sum_{\pi_{i}=1}^{m!}\bar{p^{\left(\tau \right)}}\left(\pi_{i}\right)\ln\left(\bar{p^{\left(\tau \right)}}\left(\pi_{i}\right)\right), \end{array}$$

where $\bar{p^{\left(\tau \right)}}\left(\pi_{i}\right)=1/\tau \sum_{k=1}^{\tau }p_{k}^{\left(\tau \right)}\left(\pi_{i}\right)$ with *p*_*k*_^(*τ*)^(*π*_*i*_) representing the relative frequency of the permutation pattern or dispersion pattern *π*_*i*_ in the time series *y*_*k*_^(*τ*)^.

## 4. Experimental Results

In this section, we conduct a series of comparative experiments. Comprehensive analyses of multiscale entropy values between male and female groups in the left and right hands are presented, respectively, and then the statistical difference analysis is adopted to evaluate the statistical significance of gender difference.

For each entropy measure, the mean ± SD of the MSE results is presented across all male and female subjects. The normality of the entropy results distributions is determined by the Shapiro–Wilk test. It is shown in subsequent analysis that for each entropy measure, the values in each group comply with normal distribution. On this basis, an independent two-sample *t*-test is conducted to determine the significance of differences among male and female groups. All statistical analyses are conducted using the MATLAB software, and without loss of generality, statistical significance is set to be *p* < 0.05.

### 4.1. RC_MSE_ Results

**Figure 3:**
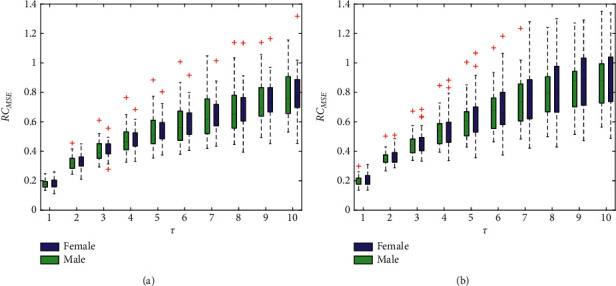
RC_MSE_ under different scales for the left hand (a) and right hand (b). The boxes show the data between the 25th and 75th percentiles, and the middle line inside each box is the median MSE value at a specified scale.

Figure 3 shows the results of RC_MSE_ measured in the left and right hands. Roughly speaking, as the scale increases, a general trend of increase regardless of the gender is revealed in the entropy values and thus the complexity of the wrist pulse signal for both hands. Moreover, the higher the scale is, the higher the complexity and variability are. A closer look at Figure 3 indicates that in the left hand, the interquartile range of entropy values for males is larger than that of females, whereas the opposite is true for the right hand. Although the actual entropy value is numerically small and the gap is very little, the median of the right hand in each group is still slightly larger than that of the left hand across all scales. Therefore, it can be concluded that compared to the left hand, the gender is more distinguishable using the right hand. However, there are some outliers found in the measurements, probably due to the hard threshold function in the computation of the SaEn.

Statistical indicators are evaluated in Table 2 for further quantitative comparison, where data are expressed as mean ± SD. We can see that there is no significant difference between genders across all scales1, although the right hand is demonstrated to be more effective than the left hand.

### 4.2. RC_MFE_ Results

**Figure 4:**
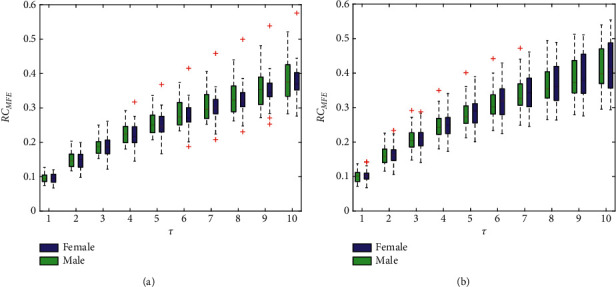
RC_MFE_ under different scales for the left hand (a) and right hand (b). The boxes show the data between the 25th and 75th percentiles, and the middle line inside each box is the median MFE value at a specified scale.

Similarly, the results of RC_MFE_ in the left and right hands are illustrated in Figure 4, showing the same trend as RC_MFE_ in both complexity and variability. Fortunately, the FuEn is more stable than the SaEn because of the more smooth growth on the scale without any outliers. The reason is that the soft and continuous boundaries used in FuEn computation enable stronger relative consistency. This confirms the FuEn measure is a better choice in follow-up pulse analysis and diagnosis research.  Table 3 tabulates the statistical indicators as well. These observations are consistent with the SaEn's.

### 4.3. RC_MPE_ Results

**Figure 5:**
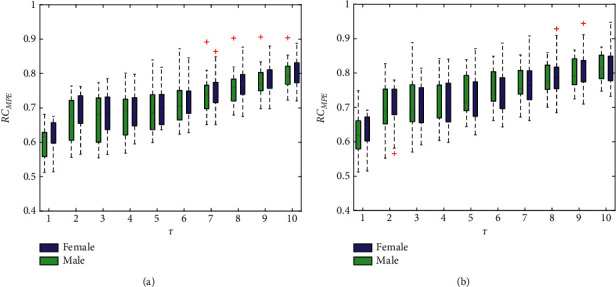
RC_MPE_ under different scales for the left hand (a) and right hand (b). The boxes show the data between the 25th and 75th percentiles, and the middle line inside each box is the median MPE value at a specified scale.

Figure 5 shows the results of RC_MPE_ in the left and right hands. In contrast, the PeEn is more robust than the two above entropy measures with a smaller fluctuation range and thereby can be provided as an alternative in subsequent diagnosis and analysis. The results of statistical analysis are shown in Table 4. Consistent with those obtained in previous studies, there is no observation of significant gender differences.

### 4.4. RC_MDE_ Results

**Figure 6:**
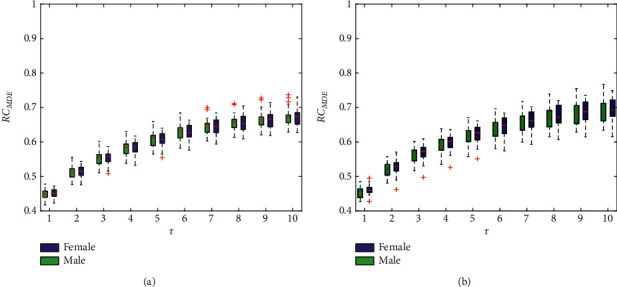
RC_MDE_ under different scales for the left hand (a) and right hands (b). The boxes show the data between the 25th and 75th percentiles, and the middle line inside each box is the median MDE value at a specified scale.

Figure 6 shows the results of RC_MDE_ in the left and right hands. As seen from the bars, the entropy fluctuation range is smaller, especially the left hand, and thus, the DiEn is most robust among the four entropy measures. This also confirms that the dispersion entropy is more robust in the study. Thereby, dispersion entropy should be the first choice in subsequent diagnosis and analysis, among TCM practitioners. The results of statistical analysis are shown in Table 5. Again, consistent with those obtained in previous studies, there is no observation of significant gender differences.

As can be seen, the results obtained with these four entropy measures are in good correspondence. In summary, two important facts can be determined. (a) For both genders, the values of the right hand exhibit slightly higher than that of the left hand, but the gap is very small with no statistical difference between left and right hands. (b) No matter which entropy measure is used, there are no overall significant gender differences over all scales. Further comparison with normal ECG signals and previous researches related to gender differences of pulse signals will be discussed in depth in the next section.

## 5. Discussion

In the present study, we assess gender differences in healthy Chinese male and female groups with four complementary entropy measures. For detailed comparative analysis, both single and multiscale sample entropy algorithms are implemented. In contrast to the previous conclusion drawn in ECG, the primary findings are that the observed difference in terms of entropy measure between males and females is not significant overall.

As stated in the Introduction, the entropy measure has also been used to distinguish between normal and control groups (different patients). Due to the different groups involved, this study cannot be directly compared. Instead, the following analysis and comparison of the research results will be discussed from three aspects: (1) the gender difference of ECG signals in normal people with respect to entropy measure, (2) the gender difference of pulse signals in existing studies, and (3) the limitations of this study. Unless otherwise specified, men and women mentioned below refer to healthy adults.

### 5.1. Comparison with Gender Difference in ECG Signals

For long-term ECG analysis, it is generally accepted that gender differences are statistically found in healthy people with respect to entropy measures. With ApEn measures, several previous works of literature [17–19] have presented similar results of higher ApEn values for women than men. It is reasonably hypothesized that these differences are due to the fact that women generally live longer and suffer from cardiovascular disease later than men [17]. On the other hand, the short-term ECG signal does not clearly show the gender difference. With multiscale sample entropy measures (only two scales of 1 and 2), it is demonstrated that there are no meaningful gender differences in short-term (5-minute) HRV for any indices [53]. Subsequently, using more multiple complexity measures [20], such as compression entropy, and by means of the detrended fluctuation analysis, the dependence on gender for some indices is proven in young subjects (25 − 34 years). However, the gender influences are considerably weaker than the age influences.

Therefore, from the above ECG signal analysis, we can see that the ECG signal is able to identify the gender on some complexity measures. Although similar to it, the results in this paper show that the pulse signal has no ability to distinguish between genders. The reason for this inconsistency may be explained as follows:

  The pulse is measured at the wrist instead of the vicinity of the heart. During the blood transfer from the heart to the wrist, many factors, such as the thickness and elasticity of vessel walls, the blood composition, and the skin conditions, will ultimately influence the fluctuations. Consequently, Kaplan [54] conjectures that the interaction of many cardiovascular feedback loops, as well as random environmental influences, may eliminate detectable traces of nonlinearity, even though the time series is caused by nonlinear mechanisms.
  On the other hand, it is inconclusive whether the normal pulse signal is nonlinear in terms of signal characteristics. With the Delay Vector Variance method [55], Yan et al. [56] examine the nonlinearity of the wrist pulse signals between the healthy group and the coronary heart disease group. Their findings report that in the coronary heart disease group, most of the wrist pulse signals (80% of 59) are nonlinear, while the wrist pulse signals of the healthy group (76% of 29) are commonly linear. But the mechanism of difference of wrist pulse's nonlinearity between two groups remains unclear.
  As mentioned in a previous study [20], the gender differences are not strong for the short-term ECG signal. In our study, the recording sample is about 2000, which may reinforce our primary findings. Due to the short data length, the nonlinear difference may be ambiguous.

It should be noted that the entropy is only one measure of complexity; no significant gender difference in entropy measure does not mean that there is no difference in complexity. As pointed out in [54], there is the possibility of gender differences in terms of complexity between healthy male and female subjects in some other nonlinear dynamics, which is just not directly supported by the used data and employed statistics. Therefore, to fully determine gender differences for pulse signals, a more thorough complexity analysis is required in the future.

### 5.2. Comparison with Gender Difference in Existing Pulse Signals Analysis

Compared with ECG signals, there are relatively few studies on gender differences of wrist pulse signals in healthy individuals. The research focus of existing work is the optimization of time-domain waveform parameters or frequency-domain spectrum parameters, not involved with complexity statistics.

Some findings have suggested the gender difference in healthy people is present in arterial pulses. For example, King et al. [57] examine the radial pulse characteristics for a sample of healthy subjects. The characteristics used include presence at the three TCM locations (Cun, Guan, Chi), depth (superficial, middle, deep), relative force, and width, most of which are qualitative measures. Although in terms of the overall pulse force, male pulses are more forceful than female pulses, the study provides limited support for TCM assumptions concerning gender-based differences in these pulse metrics. Yim et al. [22] investigate pulse differences according to gender and measuring positions in healthy individuals in a more objective manner. In this work, several time-domain parameters of the pulse signal are compared to confirm that the radial pulse differs significantly with respect to gender and measuring positions. Subsequently, Lee et al. [23] demonstrate highly significant differences in gender by performing the analysis of wrist pulse waves of healthy Korean adults at the three positions in time and frequency domains. They infer that the reason may be that men tend to have larger vascular diameters and higher blood velocities, which increase blood flow and blood pressure in the wrist artery.

TCM practitioners claim that there exist gender differences between men and women, and these previous studies have somewhat confirmed that. Yet in our study, there are no significant gender differences in nonlinear entropy measures. As stated before, no difference between the entropy does not mean that there is no difference between genders. Moreover, early studies have also shown different and special changes for specific diseases in the pulse spectrum analysis [14, 15]. Instead of entropy computation in time-domain, entropy analysis of frequency spectrum may reveal some new different results, which is worthwhile to study further.

### 5.3. Limitations and Future Studies

Undoubtedly, this paper is just a preliminary exploration to use wrist pulse signals to identify the gender of healthy people, and there are several limitations. First, the participants in this study are aged in their 20s, requiring more middle-aged healthy subjects. Also, the sample size of participants is relatively small (about 200). Second, the pulse signal itself exhibits intersubject variability. According to TCM theory, such variability has something to do with the collection time and seasonal changes [2]. In other words, it varies every day even for the same person according to his (or her) physical condition. In addition, we do not perform the pulse acquisition at multiple different locations, while existing studies have shown that the gender difference of different measures is related to collection location [58]. Third, the present study mainly assesses the entropy measure. As stated in [59], one single parameter cannot sufficiently describe complex physiological systems. Perhaps other complexity measures such as correlation dimension and detrended fluctuation analysis can provide more complementary information. Finally, some unmeasured variables, such as individual levels of fitness, may also have an impact on the gender differences we observed in wrist pulse signals. Additionally, the associated embedding dimension or tolerance parameters in entropy computation may be relevant.

In short, a further prospective study with a larger sample size, more multiple collection positions, and more broad healthy adults and complexity measures is needed to reinforce our conclusions described above, which is also our further study.

## 6. Conclusions

Pulse signal analysis is crucial to the objectification of TCM pulse diagnosis. The pulse signal and the ECG signal are similar and very important physiological signals. Regarding entropy measures, ECG signals have been confirmed to have gender differences for normal people, while corresponding characteristics of the pulse signal have not been studied. In this paper, a nonlinear analysis to investigate the gender difference within wrist pulse signals of healthy people for the first time is presented. We employ four kinds of entropy measures in the framework of refined composite multiscale to evaluate the wrist pulse signal. Extensive experiments and analyses are conducted to study the potential gender difference.

The results show that the wrist pulse signal is not as effective as the ECG signal to exhibit statistically significant gender differences for healthy people. It is however better to use the dispersion entropy measure for subsequent intelligent diagnosis since we experimentally demonstrate that it is the most robust and reliable entropy measure for the wrist pulse signal. Moreover, we believe that our findings will contribute to the modernization of pulse diagnosis and facilitate the development of pulse diagnosis systems.

Future research will investigate more influence factors of entropy measures that may be related to the gender difference. In addition, more extensive and comprehensive experiments are further required for a thorough evaluation of the wrist pulse signals.

## Figures and Tables

**Table 1:** Physical conditions of the subjects examined in this study.

Variable	Men	Women	*p* value
Age (year)	20.45 ± 0.63	20.39 ± 1.03	0.38
Height (cm)	177.36 ± 4.63	162.86 ± 4.25	<0.001
Weight (kg)	65.46 ± 8.28	55.14 ± 5.62	<0.001

**Table 2:** Statistical indicators of RC_MSE_.

*τ*	Left hand			Right hand		
	Male	Female	*P* value	Male	Female	*P* value
1	0.1802 ± 0.0325	0.1801 ± 0.0342	0.9911	0.2000 ± 0.0362	0.2087 ± 0.0461	0.4446
2	0.3233 ± 0.0491	0.3306 ± 0.0485	0.5859	0.3513 ± 0.0483	0.3673 ± 0.0563	0.2685
3	0.4065 ± 0.0744	0.4159 ± 0.0588	0.6102	0.4473 ± 0.0727	0.4688 ± 0.0855	0.3258
4	0.4773 ± 0.1016	0.4827 ± 0.0783	0.8825	0.5338 ± 0.1036	0.5605 ± 0.1302	0.4087
5	0.5404 ± 0.1286	0.5431 ± 0.0960	0.9293	0.6156 ± 0.1325	0.6464 ± 0.1664	0.4544
6	0.5969 ± 0.1509	0.5943 ± 0.1134	0.9435	0.6827 ± 0.1513	0.7140 ± 0.1933	0.5109
7	0.6508 ± 0.1631	0.6486 ± 0.1256	0.9566	0.7527 ± 0.1778	0.7689 ± 0.2012	0.7556
8	0.6882 ± 0.1732	0.6866 ± 0.1491	0.9703	0.7995 ± 0.1797	0.8248 ± 0.2042	0.6305
9	0.7342 ± 0.1712	0.7443 ± 0.1469	0.8169	0.8492 ± 0.1920	0.8687 ± 0.2077	0.7218
10	0.7820 ± 0.1807	0.7895 ± 0.1704	0.8761	0.8906 ± 0.1988	0.9055 ± 0.2058	0.7882

**Table 3:** Statistical indicators of RC_MFE_.

*τ*	Left hand			Right hand		
	Male	Female	*P* value	Male	Female	*P* value
1	0.0956 ± 0.0134	0.0954 ± 0.0155	0.9608	0.1008 ± 0.0176	0.1034 ± 0.0182	0.5926
2	0.1499 ± 0.0233	0.1485 ± 0.0268	0.8387	0.1624 ± 0.0296	0.1644 ± 0.0306	0.8080
3	0.1899 ± 0.0288	0.1875 ± 0.0328	0.7800	0.2086 ± 0.0361	0.2113 ± 0.0374	0.7813
4	0.2255 ± 0.0330	0.2219 ± 0.0373	0.7144	0.2488 ± 0.0410	0.2514 ± 0.0416	0.8197
5	0.2570 ± 0.0373	0.2520 ± 0.0409	0.6414	0.2837 ± 0.0460	0.2856 ± 0.0455	0.8773
6	0.2846 ± 0.0417	0.2777 ± 0.0439	0.5526	0.3135 ± 0.0507	0.3145 ± 0.0499	0.9404
7	0.3092 ± 0.0465	0.3015 ± 0.0467	0.5492	0.3403 ± 0.0554	0.3406 ± 0.0542	0.9845
8	0.3335 ± 0.0517	0.3247 ± 0.0498	0.5262	0.3657 ± 0.0595	0.3657 ± 0.0597	0.9972
9	0.3579 ± 0.0568	0.3508 ± 0.0539	0.6389	0.3895 ± 0.0629	0.3909 ± 0.0663	0.9398
10	0.3829 ± 0.0621	0.3769 ± 0.0579	0.7120	0.4155 ± 0.0664	0.4171 ± 0.0731	0.9325

**Table 4:** Statistical indicators of RC_MPE_.

*τ*	Left hand			Right hand		
	Male	Female	*P* value	Male	Female	*P* value
1	0.6022 ± 0.0480	0.6221 ± 0.0431	0.1153	0.6184 ± 0.0594	0.6325 ± 0.0486	0.3430
2	0.6648 ± 0.0632	0.6843 ± 0.0581	0.2427	0.6944 ± 0.0700	0.7077 ± 0.0594	0.4524
3	0.6675 ± 0.0679	0.6806 ± 0.0623	0.4644	0.7062 ± 0.0728	0.7107 ± 0.0640	0.8103
4	0.6770 ± 0.0639	0.6872 ± 0.0559	0.5330	0.7148 ± 0.0648	0.7174 ± 0.0676	0.8857
5	0.6926 ± 0.0588	0.7031 ± 0.0528	0.4937	0.7331 ± 0.0594	0.7342 ± 0.0671	0.9524
6	0.7119 ± 0.0549	0.7222 ± 0.0506	0.4784	0.7522 ± 0.0530	0.7531 ± 0.0656	0.9585
7	0.7351 ± 0.0521	0.7467 ± 0.0471	0.3953	0.7700 ± 0.0495	0.7709 ± 0.0636	0.9503
8	0.7561 ± 0.0489	0.7691 ± 0.0443	0.3102	0.7873 ± 0.0444	0.7874 ± 0.0612	0.9950
9	0.7760 ± 0.0451	0.7893 ± 0.0410	0.2629	0.8014 ± 0.0409	0.8028 ± 0.0586	0.9180
10	0.7955 ± 0.0417	0.8081 ± 0.0407	0.2662	0.8166 ± 0.0378	0.8173 ± 0.0540	0.9544

**Table 5:** Statistical indicators of RC_MDE_.

*τ*	Left hand			Right hand		
	Male	Female	*P* value	Male	Female	*P* value
1	0.4488 ± 0.0143	0.4510 ± 0.0133	0.5468	0.4505 ± 0.0218	0.4600 ± 0.0169	0.2773
2	0.5116 ± 0.0187	0.5136 ± 0.0167	0.6868	0.5164 ± 0.0278	0.5268 ± 0.0218	0.3324
3	0.5529 ± 0.0219	0.5539 ± 0.0193	0.8568	0.5580 ± 0.0324	0.5692 ± 0.0245	0.3585
4	0.5818 ± 0.0229	0.5837 ± 0.0210	0.7544	0.5880 ± 0.0350	0.5984 ± 0.0254	0.3162
5	0.6072 ± 0.0238	0.6068 ± 0.0221	0.9477	0.6133 ± 0.0382	0.6223 ± 0.0271	0.3243
6	0.6276 ± 0.0251	0.6264 ± 0.0236	0.8633	0.6333 ± 0.0412	0.6430 ± 0.0289	0.3204
7	0.6429 ± 0.0248	0.6424 ± 0.0247	0.9473	0.6498 ± 0.0431	0.6600 ± 0.0299	0.3193
8	0.6553 ± 0.0255	0.6557 ± 0.0259	0.9511	0.6640 ± 0.0456	0.6740 ± 0.0323	0.3572
9	0.6648 ± 0.0267	0.6659 ± 0.0260	0.8781	0.6741 ± 0.0476	0.6847 ± 0.0341	0.3488
10	0.6724 ± 0.0279	0.6743 ± 0.0275	0.8065	0.6812 ± 0.0530	0.6928 ± 0.0368	0.3536

## Data Availability

The data used to support the findings of this study are available from the corresponding author upon request.
